# Enhanced transmembrane electron transfer in *Shewanella oneidensis* MR-1 using gold nanoparticles for high-performance microbial fuel cells†

**DOI:** 10.1039/d2na00638c

**Published:** 2022-11-10

**Authors:** Yu-Jing Jiang, Su Hui, Shihao Tian, Zixuan Chen, Yifan Chai, Li-Ping Jiang, Jian-Rong Zhang, Jun-Jie Zhu

**Affiliations:** State Key Laboratory of Analytical Chemistry for Life Science, State Key Laboratory of Coordination Chemistry, School of Chemistry and Chemical Engineering, Nanjing University Nanjing 210023 PR China jianglp@nju.edu.cn jrzhang@nju.edu.cn jjzhu@nju.edu.cn

## Abstract

Low efficiency of extracellular electron transfer (EET) is a major bottleneck in developing high-performance microbial fuel cells (MFCs). Herein, we construct *Shewanella oneidensis* MR-1@Au for the bioanode of MFCs. Through performance recovery experiments of mutants, we proved that abundant Au nanoparticles not only tightly covered the bacteria surface, but were also distributed in the periplasm and cytoplasm, and even embedded in the outer and inner membranes of the cell. These Au nanoparticles could act as electron conduits to enable highly efficient electron transfer between *S. oneidensis* MR-1 and electrodes. Strikingly, the maximum power density of the *S. oneidensis* MR-1@Au bioanode reached up to 3749 mW m^−2^, which was 17.4 times higher than that with the native bacteria, reaching the highest performance yet reported in MFCs using Au or Au-based nanocomposites as the anode. This work elucidates the role of Au nanoparticles in promoting transmembrane and extracellular electron transfer from the perspective of molecular biology and electrochemistry, while alleviating bottlenecks in MFC performances.

## Abstract 2

Au nanoparticles can act as electron channels to facilitate the transmembrane and extracellular electron transfer in *Shewanella oneidensis* MR-1.

## Graphical abstract

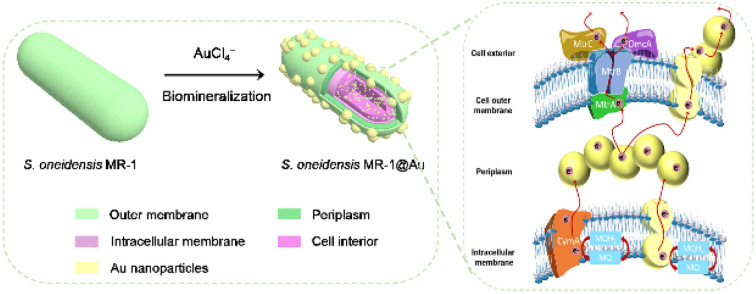

## 1 Introduction

Microbial fuel cells (MFCs), which can directly convert chemical energy stored in biodegradable organic wastes or biomass into electrical energy through microbial metabolism, are applicable for wastewater remediation, desalination, and the removal of toxic chemicals from the environment.^1–4^ However, the low power density remains a fundamental bottleneck for the practical application of MFCs and the MFC performance relies heavily on extracellular electron transfer (EET) between the intracellular respiratory chains of exoelectrogens and electron acceptors.^5–8^ Among all the exoelectrogens, *Shewanella oneidensis* is one of the well-studied exoelectrogens type strains due to their robust growth in aerobic and anaerobic environments and their abundant distribution in soil and seawater.^9^ Two EET mechanisms of *S. oneidensis* have been demonstrated through extensive research.^10^ One is indirect electron transfer mediated by endogenously secreted soluble redox molecules.^11^ Another EET mechanism is the contact-based direct extracellular electron transfer (DET), in which electrons are directly transferred to the anode *via* a number of conductive outer-membrane c-type cytochromes.^12–15^ Notably, DET efficiency is generally considered as a key factor in improving MFC performance,^16^ but is currently limited by inefficient interfacial contact between exoelectrogens and the electrode surface.^17^

In recent years, substantial efforts have been centered on improving the DET efficiency through modifying electrodes with various functional nanomaterials,^18,19^ including carbonaceous material,^20–22^ metals,^23^ metal oxides^24^ and conducting polymers.^25^ Highly conductive nanomaterials can act as electron transport channels for bacteria and thus significantly improve the EET efficiency.^26^ Moreover, the additional active sites introduced by them can improve interfacial electron transfer between bacteria and the electrode, leading to efficient biocatalysis and electrocatalysis.^27^ However, most of the bacterial cells inside the natural biofilm formed in this way are far from being functional nanomaterials, and can only transfer electrons to the electrodes through slow electron hopping of multiple redox centers between bacteria, limiting the improvement of energy output in MFCs.^28,29^ In this context, a single-bacterial surface modification strategy was proposed to construct an interconnected intact conductive layer on and across the individual cell membranes for creating highly conductive and stable catalytic interfaces for exoelectrogens and electrodes.^30–33^ Conductive polymers, including polypyrrole (PPy), polydopamine (PDA) and their composites, have been used to coat the bacterial surface to reduce charge transfer resistance.^34,35^ However, these materials were mainly attached to the outer membrane of bacterial cells and were difficult to embed into the periplasm or inner membrane. In view of this, if the transmembrane electron transport can be further facilitated, the MFC performance would be greatly improved.^36^ In addition, efficient interfacial electron transfer requires extremely close contact between the transmembrane electron transfer conduits and conductive abiotic surface.^37^ The biomineralization mechanism of exoelectrogens provides a possibility for the realization of this strategy.^16^ Several metal nanoparticles (NPs) and their complexes have been studied to improve the transmembrane electron transport efficiency and the cell viability.^38–40^ Compared with metal oxides, pure metal NPs possess the advantages of higher conductivity, higher stability, better nanostructure manipulation ability and catalytic activity.^41^

Gold nanoparticles (Au NPs) are the most stable metal nanoparticles and have widely served as an ideal anode surface modification material to improve EET efficiency and bacterial adhesion, due to their good biocompatibility, high conductivity and tunable surface charge. Au NPs can accelerate the growth of the *Shewanella oneidensis* MR-1 biofilm, and MFCs based on a carbon paper-Au anode generate 47% higher total electric charges than MFCs with a carbon paper anode.^42^ Furthermore, the coulombic efficiency and power generation could be increased with the Au density increasing on the anode surface. By depositing carbon paper with an Au thickness of 100 nm on each side, the maximum power density was enhanced by 188% and the stabilization time of maximum power generation was increased by 122%.^43^ In most cases, Au NPs are fabricated using various chemical methods under heat or sonication conditions. In contrast, Au NPs fabricated by microbial methods not only have the advantage of requiring fewer chemical reagents and reactions under mild conditions, but also have better biocompatibility and higher catalytic activity. Wu *et al.* tested biogenic Au NPs for anode modification in MFCs, which resulted in a 23% increase in maximum power density compared to a bare carbon cloth control.^44^ In previous studies, Au NPs were mainly used as electrode modification materials. Although single-bacterial surface modification technology has been introduced in MFCs, the enhancement of the power generation performance of exoelectrogens by Au NPs has not been studied and the exact location and basic role of these Au NPs in exoelectrogens remain elusive.^45^ Therefore, it is necessary to elucidate the specific mechanism by which metal NPs enhance the EET efficiency through in-depth mechanistic studies, and to design a high-performance bioanode to fundamentally address the EET limitations.

**Fig. 1:**
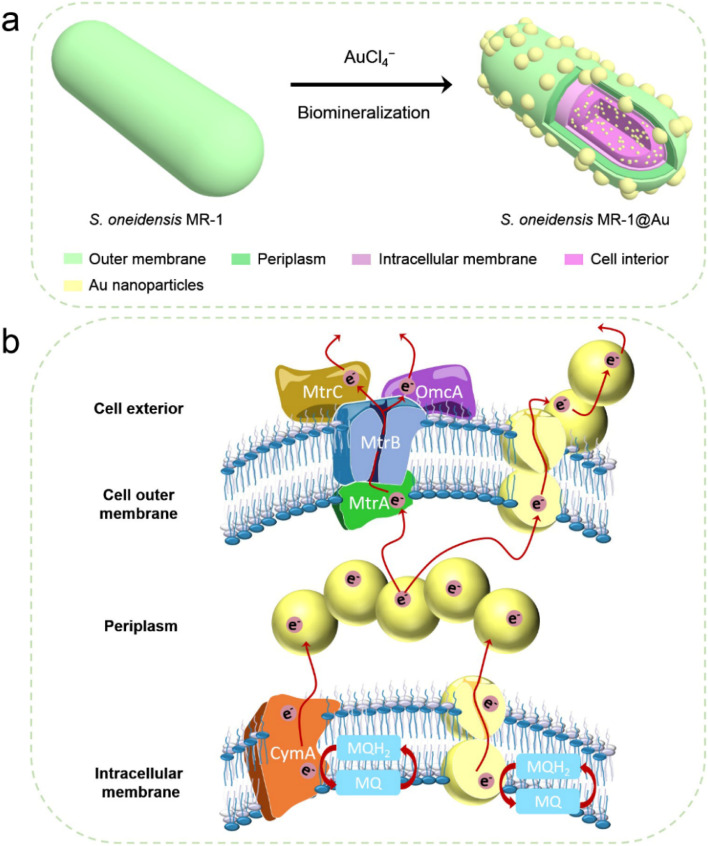
(a) Schematic illustration of the synthesis of *S. oneidensis* MR-1@Au for enhanced bioelectricity generation. (b) The hypothetical electron transfer pathway in *S. oneidensis* MR-1@Au.

Herein, we fabricated *S. oneidensis* MR-1@Au by *in situ* biomineralization, and deeply studied the mechanism of Au NPs for the enhancement of EET efficiency from the perspective of molecular biology and electrochemistry (Fig. 1). Our systematic studies demonstrated that Au NPs not only tightly covered the bacteria surface, but were also distributed in the periplasm, cytoplasm and cell membrane. These Au NPs could act as electron conduits to provide additional electron channels for membrane cytochromes to facilitate transmembrane and extracellular electron transport, thereby enhancing overall MFC performance. This work not only relieves the bottleneck of MFC performance, but also provides guidance for the design of high-performance bioanodes.

## 2 Results and discussion

### 2.1 Assembly of *S. oneidensis* MR-1@Au

**Fig. 2:**
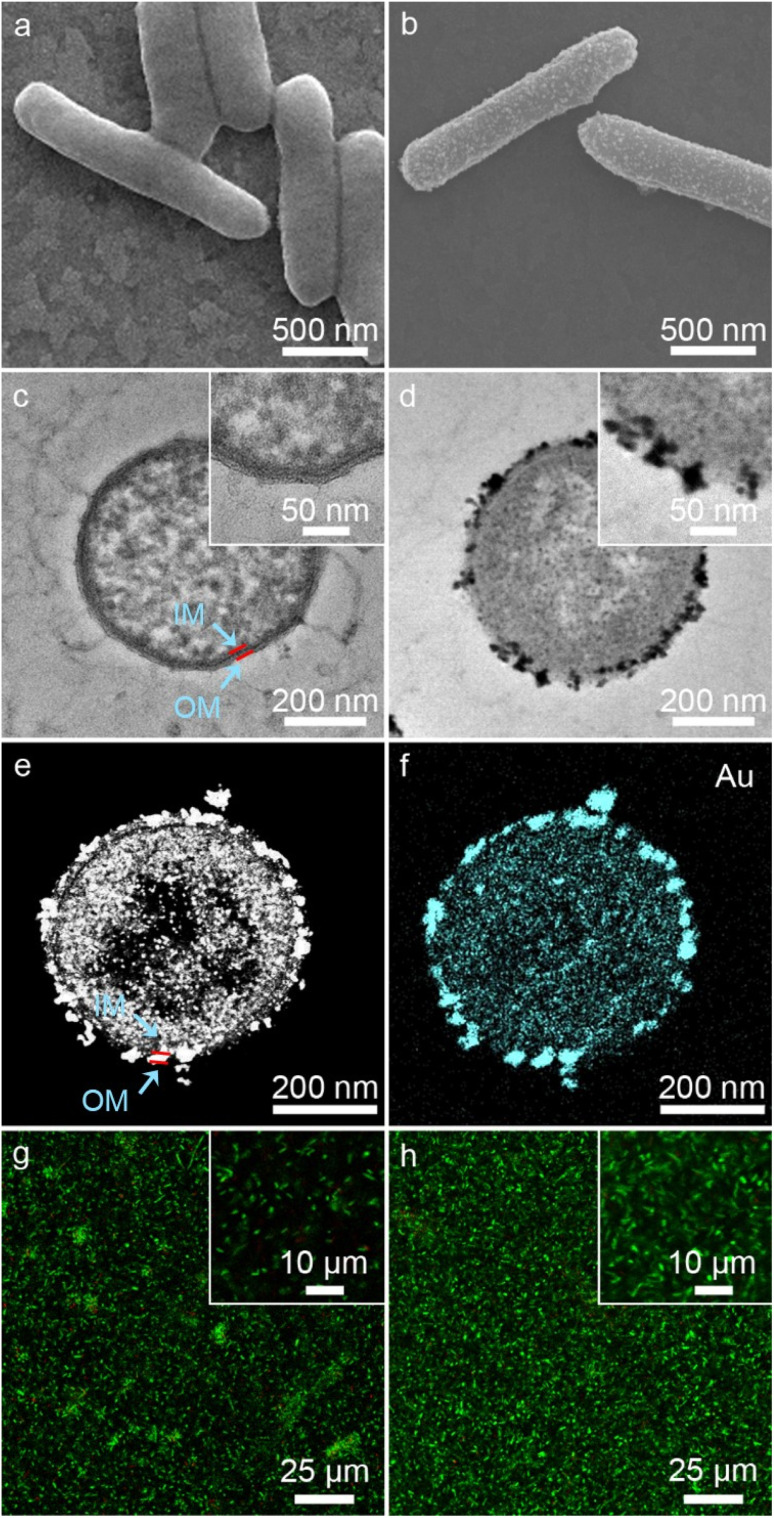
(a and b) SEM images of *S. oneidensis* MR-1 (a) and *S. oneidensis* MR-1@Au (b). (c and d) TEM images of cross-section slices of *S. oneidensis* MR-1 (c) and *S. oneidensis* MR-1@Au (d). The insets of the image (c and d) are the corresponding high-magnification TEM images. (e) HAADF-STEM image of cross-section slices of *S. oneidensis* MR-1@Au. (f) Element mapping image of cross-section slices of *S. oneidensis* MR-1@Au. OM: outer membrane of *S. oneidensis* MR-1 and IM: inner membrane of *S. oneidensis* MR-1. (g and h) CLSM images of *S. oneidensis* MR-1 (g) and *S. oneidensis* MR-1@Au (h), respectively. The insets of the image (g and h) are the corresponding high-magnification CLSM images.

As shown in Fig. 2a, native *S. oneidensis* MR-1 cells were rod-shaped with a relatively smooth surface. *S. oneidensis* MR-1@Au were fabricated using an *in situ* biomineralization synthesis strategy (Fig. 1a).^46^ By simply adjusting the polymerization time (the optimum polymerization time is 16 h), a uniformly covered Au nanoshell was assembled on the cell surface (Fig. 2b and S1†). To evaluate the spatial distribution of Au NPs within an individual bacterium, the transmission electron microscope (TEM) image, high-angle annular dark field-scanning transmission electron microscope (HAADF-STEM) image and energy dispersive X-ray (EDX) elemental mapping image of *S. oneidensis* MR-1@Au were further recorded. The TEM images of cross-section slices of pristine *S. oneidensis* MR-1 cells showed a relatively smooth profile and uniform contrast, whereas distinct black spots were found in *S. oneidensis* MR-1@Au (Fig. 2c, d and S2†). Strikingly, abundant Au NPs were aligned in the periplasm and cytoplasm, and even embedded in the cell membrane (Fig. 2e and S3†). The size of these transmembrane and extracellular NPs was approximately 10–30 nm, while the intracellular NPs had a diameter between 5 and 10 nm. Due to the limitation of intracellular space, nanoparticles formed intracellularly are smaller in size than those formed extracellularly.^12^ A possible mechanism for this phenomenon is that AuCl_4_^−^ diffused into the bacteria and was then reduced *in situ* by electrons generated by metabolism, resulting in the formation of Au NPs.^47^ Elemental mapping revealed a uniform distribution of the Au element in and across the cell membrane (Fig. 2f). Moreover, the nanoparticles were characterized by X-ray diffraction (XRD) analyses (Fig. S4†). The characteristic peaks in the XRD spectrum of *S. oneidensis* MR-1@Au can correspond to the (111), (200), (220) and (311) planes of Au NPs, proving the formation of Au NPs on *S. oneidensis* MR-1.^48^ This result indicated that the nanoparticles present on the cell surface were indeed Au NPs. All these results demonstrated the successful assembly of *S. oneidensis* MR-1@Au.

Moreover, based on the differential permeability between an intact or compromised cell membrane, we assessed the cell viability after biomineralization. While propidium iodide (PI, red fluorescence) penetrates into cells with damaged membranes exclusively and indicates the dead cells, SYTO 9 (green signal) can stain both living and dead cells. The confocal laser scanning microscope (CLSM) images of *S. oneidensis* MR-1 and *S. oneidensis* MR-1@Au both showed strong green fluorescence and slight red fluorescence (the proportion of red fluorescence in both is less than 5%, Fig. S5a†), indicating that neither the biomineralization process nor the Au NPs were detrimental to bacterial activity (Fig. 2g and h). Although some studies have proved that Au NPs can impair bacterial activity by altering the permeability of cell membrane, the concentration of Au NPs used in these studies were much higher than that used in our study. In addition, the difference in the synthesis method and particle size is also a main reason for cytotoxicity. Considering the heavy metal tolerance of *Shewanella* and the biomineralization synthesis method, it is not surprising that *S. oneidensis* MR-1@Au maintained such high cell viability.^49^ We further explored the effect of Au NPs on bacterial activity after 120 h cultivation. Compared with the initial CLSM image (Fig. 2g), the CLSM image of native bacteria showed more red fluorescence (Fig. S6a†), indicating an increased percentage of dead cells. In contrast, the functionalized bacteria retained higher activity (Fig. S6b†). The proportion of red fluorescence in native bacteria after 120 h cultivation is 24.3 ± 6.9%, which is 4.5 times higher than that in *S. oneidensis* MR-1@Au (Fig. S5b†). These results suggested that Au NPs are beneficial for maintaining bacterial viability during long-term operation. In addition, the cell growth curves showed that the biomineralization of Au NPs significantly prolonged the lag phase of *S. oneidensis* MR-1 and the modified cells could retain the capability of dividing themselves under aerobic conditions (Fig. S7†).^38^

### 2.2 Electricity generation capability of *S. oneidensis* MR-1@Au

**Fig. 3:**
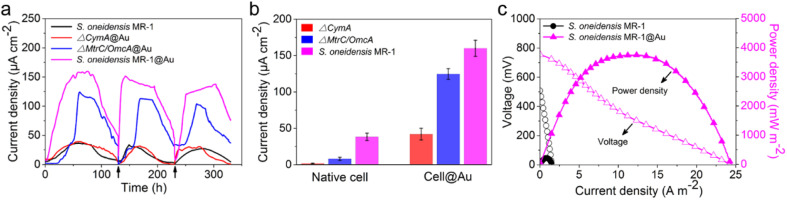
(a) Time profile of electricity generation of different bioanodes in electrochemical half-cells. Arrows represent the replacement of a fresh medium. (b) Current output of native or functionalized cells (*n* = 3) in electrochemical half-cells. Error bars represent standard error (s.e.) determined by three independent experiments. (c) Polarization (hollow symbols) and power density output curves (solid symbols) of different bioanodes in MFCs.

After successful assembly of *S. oneidensis* MR-1@Au, the output current density of the functionalized bacteria was evaluated with a three-electrode system in an electrochemical half-cell.^50^ The current output of *S. oneidensis* MR-1@Au increased continuously with incubation time and reaches a nearly constant value within 45 h (Fig. 3a), indicating the successful establishment of a stable biofilm. It then dropped sharply after 35 hours of stable operation, which can be attributed to the substrate consumption and metabolite accumulation. After the replacement of the fresh medium (Fig. 3a, *t* = 130 min and *t* = 230 min), the maximum current density can be restored to a parallel level. Strikingly, *S. oneidensis* MR-1@Au delivered a current density approximately 4.3 times higher than that of the native bacteria (159 μA cm^−2^*vs.* 37 μA cm^−2^, Fig. 3a), suggesting that the Au NPs formed by *in situ* mineralization could significantly improve the electricity generation. Moreover, the electrochemical half-cells with dead *S. oneidensis* MR-1@Au did not deliver a significant current output, indicating that the electrons were only derived from the bacteria (Fig. S8†).

Considering that *S. oneidensis* MR-1 are not easily apt to generate bionanowires to achieve electron transfer in nutrient-rich environments, membrane cytochromes might play a main role in EET. In such a process, the electrons transferred extracellularly mostly originate from lactate oxidation by lactate dehydrogenase.^51,52^ The electrons from menaquinone are hopping from the CymA cytochromes redox centers on the inner membrane to the periplasm. Subsequently, the electrons are further transferred through outer membrane proteins MtrA, MtrB, MtrC, and OmcA to the electrode surface (Fig. 1b). To further explore the role of Au NPs in transmembrane and extracellular electron transport, we also investigated the corresponding mutants *ΔCymA* and *ΔMtrC/OmcA*. As expected, the disruption of CymA or MtrC/OmcA greatly suppressed (by over 95% and 81%, respectively) the current output of the native *S. oneidensis* MR-1 (Fig. 3b). It has been reported that Au NPs could participate in catalyzing the oxidation of organics and repairing cell damage in electron transfer to some extent.^46^ Compared with *ΔCymA*, which can hardly generate current, the current of *ΔCymA*@Au can be recovered to 42 ± 8 μA cm^−2^, indicating that Au NPs played a role in electron transport across the inner membrane similar to CymA (Fig. 3b). Strikingly, the current of *ΔMtrC/OmcA*@Au reached 125 ± 7 μA cm^−2^, which was 3.4 times higher than that of native *S. oneidensis* MR-1, proving that the Au NPs embedded in the periplasm and outer membrane made a major contribution to transmembrane electron transfer. Taken together, these results demonstrated that Au NPs could act as electron conduits to provide additional electron channels for membrane cytochromes to facilitate transmembrane and extracellular electron transport.

To probe the power output of *S. oneidensis* MR-1@Au, we constructed a double-chamber MFC and measured the polarization curves when the MFC was stably discharged (Fig. 3c). Impressively, the maximum power density of the MFC with *S. oneidensis* MR-1@Au bioanode reached up to 3749 mW m^−2^, which was 17.4 times higher than that with the native *S. oneidensis* MR-1 (216 mW m^−2^). This superior power density is clearly higher than those of previously reported MFCs using Au and Au-based nanocomposites as anodes (Table 1). All these results demonstrate that the Au NPs not only improved the EET efficiency of the individual cell but also facilitated electron transfer across the biofilm, thereby enhancing the power output.

**Table 1:** Comparison of the performance of previous MFCs using Au and Au-based nanocomposites as anodes

Electrode substrates	Anode materials	Microbe type	Power density (mW m^−2^)	Ref.
Carbon paper	CNT/Au/TiO_2_	*E. coli*	2.4	53
Carbon felt	MWCNT-Au-Pt/osmium redox polymer	*Gluconobacter oxydans*	32.1	54
Carbon cloth	BioAu/MWCNT	Mixed bacteria	178.34 ± 4.79	44
Carbon paper	Au	Mixed bacteria	346	55
Carbon paper	Au	Mixed bacteria	461.6	43
Carbon paper	G/Au	*Shewanella oneidensis*	508	56
Carbon cloth	Au@PANI	*E. coli*	804 ± 73	57
Carbon paper	Au	Mixed bacteria	990	58
Carbon cloth	—	Au and Fe_3_O_4_-coated *Shewanella oneidensis*	1792	45
Carbon felt	—	*E. coli*@Au_1_@CdS_1_	2300.4	40
—	Fe_3_O_4_/Au NCs-3DGF	*Shewanella oneidensis*	2980 ± 54	59
**Carbon felt**	**—**	***S. oneidensis* MR-1@Au**	**3749**	**This work**

### 2.3 Mechanism investigation

**Fig. 4:**
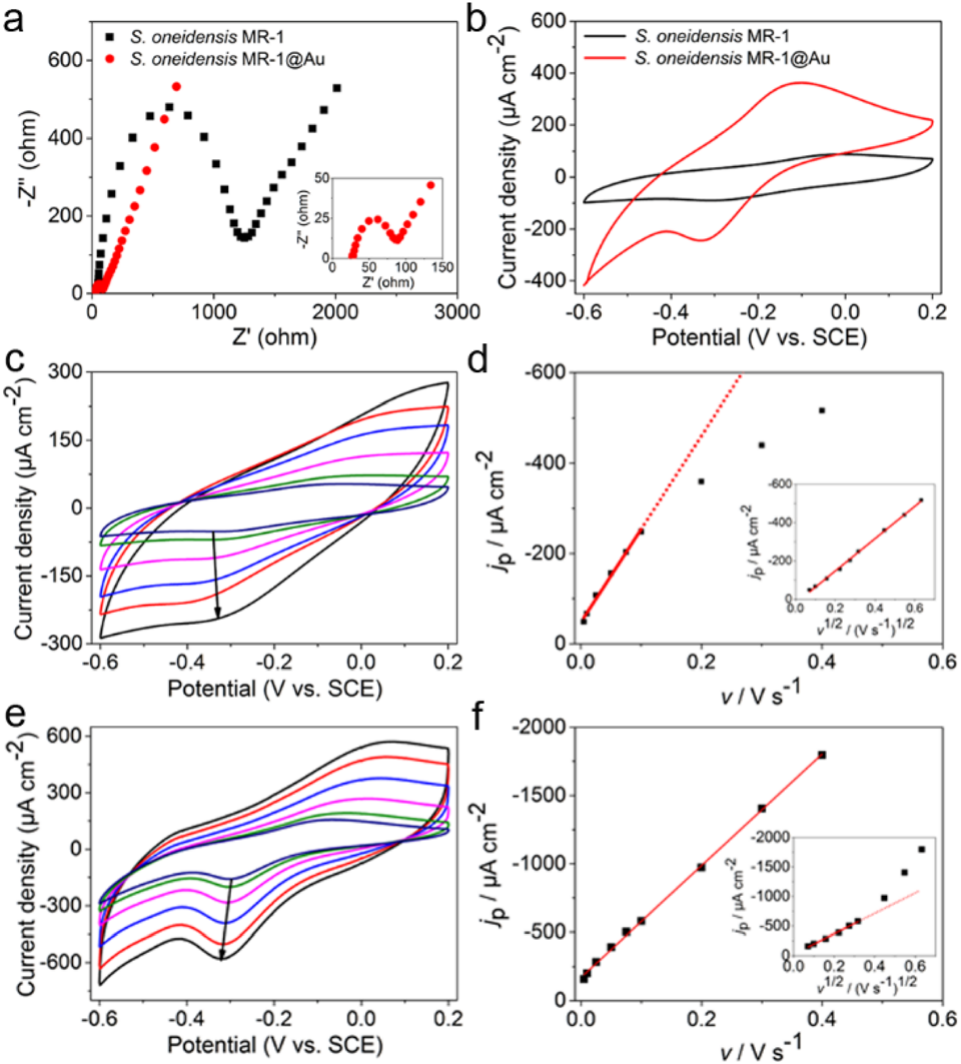
(a) Nyquist plots of electrochemical impedance spectroscopy of *S. oneidensis* MR-1 and *S. oneidensis* MR-1@Au bioanodes in 10 mM Fe(CN)_6_^3+^/Fe(CN)_6_^4+^ containing 100 mM KCl. (b) CV curves of *S. oneidensis* MR-1 and *S. oneidensis* MR-1@Au in electrochemical half-cells without a carbon source at a scan rate of 5 mV s^−1^. Cyclic voltammograms of *S. oneidensis* MR-1 (c) and *S. oneidensis* MR-1@Au (e) biofilms at different scan rates (arrows showed scan rates at 5, 10, 25, 50, 75 and 100 mV s^−1^, respectively). Dependence of reduction current density (*j*_p_) *versus* scan rate (*v*) on *S. oneidensis* MR-1 (d) and *S. oneidensis* MR-1@Au (f) biofilms, separately; inset: linear dependence of *j*_p_*versus v*^1/2^.

To explore the reasons for the excellent performance of the *S. oneidensis* MR-1@Au bioanode, we need to further understand the role of Au NPs in the charge transfer process. EET efficiency is the primary factor that is highly associated with electricity generation and electron utilization capability.^60^ Thus, the electrochemical impedance spectroscopy (EIS) technique was employed to evaluate the interfacial charge transfer behaviors of different bioanodes in 10 mM Fe(CN)_6_^3+^/Fe(CN)_6_^4+^ containing 100 mM KCl. In the Nyquist curve, the semicircle portion at higher frequencies corresponds to charge transfer resistance (*R*_ct_) at the liquid–solid interface, which represents the resistance of electrochemical reactions on the electrode and determines the electron transfer kinetics of bioanodes.^61^ As shown in Fig. 4a, the *S. oneidensis* MR-1@Au bioanode reduced the interfacial charge transfer resistance by approximately 19.5 times (65 Ω *vs.* 1270 Ω), suggesting that Au NPs could significantly facilitate electron transport inside the biofilm and help to establish a favorable interface between the bacteria and the extracellular solid conductive surface.

Furthermore, the redox reaction kinetics at cell–electrode interfaces at the pseudo-steady state were analyzed in detail by cyclic voltammetry (CV). In native *S. oneidensis* MR-1, electron transport from the cytoplasmic membrane across the periplasm and outer membrane to the electrode is strongly dependent on the efficient pathway of cytochrome chains. Before performing the CV analyses, the medium was changed to fresh M9 buffer to remove the effect of flavin and lactate. As shown in Fig. 4b, the CV curve of *S. oneidensis* MR-1 bioanode showed a couple of peaks at −0.31 V and −0.03 V, which are relevant to the electrochemical response of outer membrane c-type cytochromes.^34,36^ Since the peak separation (Δ*E*_p_) between the oxidation and reduction peaks is inversely proportional to the electron transfer rate, a smaller Δ*E*_p_ represents an increase in the EET rate.^55^ Compared with the *S. oneidensis* MR-1 bioanode, the Δ*E*_p_ of the *S. oneidensis* MR-1@Au bioanode was significantly reduced (0.28 V *vs.* 0.22 V), illustrating that Au NPs could obviously facilitate the DET efficiency and promote the electron exchange at the electrode surface. To get deeper into the redox reaction kinetics at the cell–electrode interface, we collected the CVs of different bioanodes at a series of scan rates (from 5 to 400 mV s^−1^). It is noted that the peak current of the *S. oneidensis* MR-1 bioanode depended linearly on the square root of the scan rates, indicating that the redox reaction of c-type cytochromes of *S. oneidensis* MR-1 was a typical diffusion-controlled process (Fig. 4c and d). In contrast, the peak current of the *S. oneidensis* MR-1@Au bioanode showed a good linear relationship with the scan rate, implying that the surface-controlled electron transfer process was prominent (Fig. 4e and f).^62^ These results further confirmed the promoted EET after the modification of Au NPs on the cell surface.

### 2.4 Biomass and activity determination of the bioanode

**Fig. 5:**
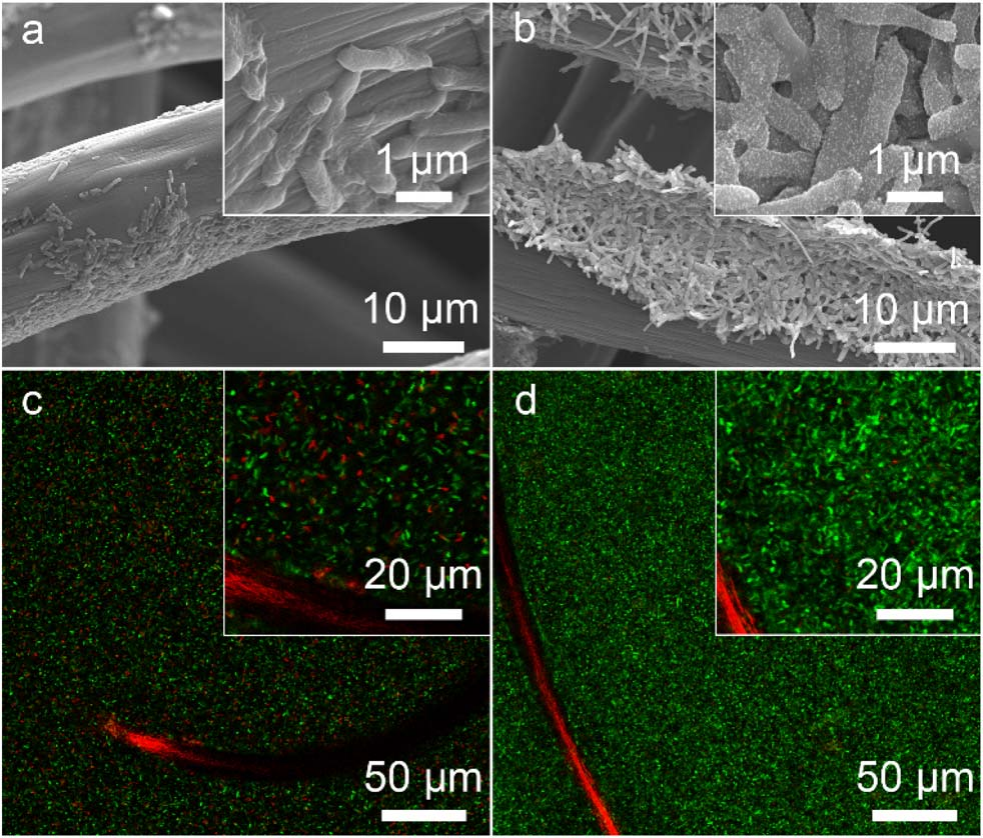
SEM (a and b) and CLSM (c and d) images of *S. oneidensis* MR-1 (a and c) and *S. oneidensis* MR-1@Au (b and d), respectively. The insets of the image (a–d) are the corresponding high-magnification images.

The bacteria-loading amount on the electrode surface and cell viability are also important factors affecting the MFC performance. We investigated the morphology of the bioanode when the MFCs reached the highest voltage. A compact biofilm consisting of densely packed bacteria was found in the *S. oneidensis* MR-1@Au bioanode (Fig. 5a and b), whereas only a few native *S. oneidensis* MR-1 were attached to the carbon felt anode. Furthermore, the biomass of these bioanodes was quantitatively determined by using a Detergent Compatible Bradford Protein Assay Kit. As expected, the bacteria loading mass of the *S. oneidensis* MR-1@Au bioanode (276.2 ± 21.2 μg cm^−2^) was significantly higher than that of the native *S. oneidensis* MR-1 bioanode (158.5 ± 36.7 μg cm^−2^). This biomass determination result coincided well with that from scanning electron microscope (SEM) analysis, indicating that the presence of the Au NPs is beneficial to the formation of a dense biofilm. We have also evaluated the cell viability of these bioanodes in MFCs over 120 h operation (Fig. 5c and d). The percentage of dead bacteria on the surface of *S. oneidensis* MR-1 is 41.7 ± 11.2%, which is 8.9 times higher than that of *S. oneidensis* MR-1@Au (Fig. S9†), proving that Au NPs exhibit terrific biocompatibility which helps the bacterial proteins to retain their native structure and enzymatic activity and thus increase bacterial stability in long-term operation. These results demonstrated that the *S. oneidensis* MR-1@Au bioanode exhibits higher EET efficiency, better bioactivity and more biological attachment, which enables the rapid transfer of electrons from the bacteria to the electrode and results in an enormous increase in MFC performance.

## 3 Conclusions

In summary, a *S. oneidensis* MR-1@Au bioanode was constructed by *in situ* biomineralization. Transmembrane electron channels formed by Au NPs enabled an extraordinarily enhanced electron transfer compared with that in native bacteria, thus exhibiting promising applications in MFCs. Both the greatly enhanced current output in electrochemical half-cells and the power output in MFCs demonstrated the significant improvement in bioelectricity production. Notably, the maximum power density of the *S. oneidensis* MR-1@Au bioanode reached up to 3749 mW m^−2^, reaching the highest performance yet reported in MFCs using Au or Au-based nanocomposites as the anode. This work provides proof of mechanism for the enhancement of Au-facilitated bioelectricity generation from the perspectives of molecular biology and electrochemistry, and points out the direction for the construction of a high-performance bioanode in MFCs.

## 4 Experimental methods

### 4.1 Chemicals and materials

*S. oneidensis* MR-1 was purchased from the American Type Culture Collection (Manassas, VA, USA). All mutants of *S. oneidensis* MR-1 were kindly provided by Prof. Yang-Chun Yong (Jiangsu University). Carbon felt was purchased from Beijing Jinglong Special Carbon Technology Company (Beijing, China). Luria–Bertani (LB) broth was obtained from Haibo Biological Reagent Company (Qingdao, China). KH_2_PO_4_, Na_2_HPO_4_, NaCl and MgSO_4_ were provided by Nanjing Reagent Company (Nanjing, China). HAuCl_4_ was obtained from Shanghai Reagent Company (Shanghai, China). Ultrapure water with a resistance of 18.2 MΩ was used for all experiments.

### 4.2 Microbe culture and assembly of functionalized bacteria

The native *S. oneidensis* MR-1 and the corresponding mutants *ΔCymA* and *ΔMtrC/OmcA*, with an inoculation of 5%, were inoculated in a Luria–Bertani (LB) broth growth medium and incubated aerobically at 30 ± 1 °C for 24 h in an orbital shaker (150 rpm). The bacterial cells harvested by centrifugation (5500 rpm, 5 min) were then resuspended in 15 mL M9 buffer (22 mM KH_2_PO_4_, 42 mM Na_2_HPO_4_, 85.5 mM NaCl, and 1.0 mM MgSO_4_) containing 18 mM lactate for further electrochemical experiments.

Prior to the formal biomineralization of *S. oneidensis* MR-1@Au, the domestication experiments were conducted to improve the tolerance of *S. oneidensis* MR-1 to Au NPs. To be specific, we first added chloroauric acid solution to three tubes containing 15 mL LB broth to make the final concentration of chloroauric acid to 0.1, 0.2 and 0.3 mM, respectively. After 24 h of cultivation, the first two groups had obvious turbidity, while the last group grew slowly. Subsequently, bacteria cultured in 0.2 mM chloroauric acid were inoculated into three tubes containing 15 mL LB broth, and chloroauric acid solution was added to make the final concentration of chloroauric acid be 0.2, 0.4 and 0.6 mM, respectively. Following the above steps, the well-grown bacteria in the experimental group after culturing for 24 h were inoculated into LB broth with a gradually increased concentration of chloroauric acid. Finally, the bacteria cultured in the medium containing 1.5 mM chloroauric acid were used as the experimental group. In addition, the same acclimation experiments were also performed before the formal biomineralization of *ΔCymA*@Au and *ΔMtrC/OmcA*@Au.

Functionalized bacteria were prepared by biomineralization using the following steps. First, *S. oneidensis* MR-1 cells, with an inoculation of 5%, were cultured aerobically for 24 h and harvested by centrifugation (5500 rpm, 5 min). The cells were washed with sterile water three times and resuspended in M9 buffer solution containing 18 mM lactate. Subsequently, nitrogen was bubbled for 15 min to remove oxygen, and chloroauric acid ([AuCl_4_^−^] = 1.5 mM) was then added to the cell suspension for *in situ* biomineralization of Au NPs. After 16 hours of anaerobic incubation, the cells were washed with ultrapure water two times to remove excess precursor and stored for further use. Moreover, the formation of *ΔCymA*@Au and *ΔMtrC/OmcA*@Au was achieved following the same procedure.

### 4.3 Assembly of bacteria/carbon felt electrodes

The native bacteria and cell@Au were concentrated by centrifugation (5500 rpm, 5 min), respectively. Subsequently, the carbon felt (1 × 1 × 0.1 cm, projected area 2 cm^2^) was immersed in a concentrated bacterial solution (1 mL) and incubated aerobically at 30 °C for 24 h, allowing bacteria to naturally deposit and attach to the carbon felt to form a stable biofilm.

### 4.4 Half-cell microbial fuel cell experiments

Half-cell microbial fuel cell experiments were carried out with a CHI 760E electrochemical workstation (Chenhua, Shanghai, China).^12,50^ The prepared bacteria/carbon felt electrode was used as the working electrode. A graphite rod electrode (6 mm in diameter) and saturated calomel electrode were used as the counter electrode and the reference electrode, respectively. Native or functionalized bacteria were used as the inoculum. All electrochemical experiments in half-cell microbial fuel cells were anaerobically performed at a constant potential of 0.2 V at 30 ± 1 °C. EIS measurement was conducted in a three-electrode system at a potential amplitude of 5 mV in a frequency range between 100 kHz and 1 mHz. Before the EIS measurement, we used glutaraldehyde to fix the prepared bacteria/carbon felt electrodes for 2 h to ensure the integrity of the bacterial structure during the measurement process.^34^ In addition, the voltage used in EIS is the open circuit potential (OCP). The solution used for EIS contains 10 mM Fe(CN)_6_^3+^/Fe(CN)_6_^4+^, 10 mM PBS and 100 Mm KCl.^45^ CV was performed using a potentiostat (CHI 760E, Chenhua, Shanghai, China) at a series of scan rates from 5–400 mV s^−1^ over a range between −0.6 V and 0.2 V in the same three-electrode system. Before performing the CV analyses, the medium was changed to fresh M9 buffer to remove the effect of flavin and lactate.

### 4.5 MFC construction and operation

An H-shaped MFC was constructed by using two cylindrical plexiglass bottles, which served as an anode chamber and a cathode chamber, each with an operating volume of 120 mL. The two chambers were separated by a proton exchange membrane (PEM) Nafion 117 separator and connected to an external resistance of 1000 Ω. The anode chamber was equipped with native or functionalized bacteria and fed with M9 buffer solution containing 18 mM lactate and 5% LB broth. The prepared bacteria/carbon felt electrode was used as the bioanode. The cathode was equipped with a graphite rod (6 mm in diameter) and filled with 50 mM potassium ferricyanide solution in 100 mM phosphate buffer (pH 7.0). Before the test, the anodic solution was purged with pure nitrogen gas for 30 min to remove the dissolved oxygen. The MFCs were operated at 30 ± 1 °C and all runs were conducted three times. The polarization curves were obtained by linear sweep voltammetry during the stable power production stage and the anode solution was refreshed when the voltage dropped below 50 mV.

### 4.6 Electron microscopy

Morphology of bacteria and materials was characterized by using a JEOL/JEM-2800 scanning electron microscope at an acceleration voltage of 10 kV and a JEOL/JEM-2100 transmission electron microscope at an accelerating voltage of 200 kV. Prior to the SEM experiments, the bacteria were suspended in a 2.5% glutaraldehyde solution (prepared in 10 mM phosphate buffer) for 3 h and sequentially dehydrated using gradient ethanol/water solutions (25%, 50%, 75%, 95% and 100%).^63^ The sample was dried and sputter-coated with platinum for further SEM imaging. Prior to the TEM experiments, the bacteria were fixed by using 2.5% glutaraldehyde solution overnight at 4 °C and 1% osmic acid solution for 2 h. After fixation, the bacteria were washed three times with PBS buffer solution and dehydrated using a gradient concentration of ethanol (30, 50, 70, 80, 90, 95% and 100%). The bacterial pellet was finally embedded in resin and polymerized in an oven at 70 °C overnight. Ultrathin sections of 70–90 nm were cut by using an Ultratome and deposited on carbon coated copper grids for TEM imaging. In addition, cell viability was studied by using a confocal laser scanning microscope (Leica TCS SP8, Germany).^64^ The bacteria and the anode biofilm were rinsed with M9 buffer solution and stained with SYTO 9 and PI mix solution. Cells with intact or compromised membranes were differentiated based on differential permeability of the fluorescent dye.^65^ In living bacteria, SYTO 9 will emit strong green fluorescence. In dead bacteria, PI will bind to DNA and emit red fluorescence.

### 4.7 Structural characterization and biomass quantification

XRD analysis was performed on a diffractometer (XRD-6100, Shimadzu, Japan) with Cu Kα radiation (*λ* = 1.5405 Å). The biofilm biomass was estimated from the amount of total protein on the electrode. The electrode after an MFC cycle was immersed in 1.0 M NaOH solution and sonicated to break the biofilm from the electrode. Subsequently, protein measurements were performed with a Detergent Compatible Bradford Protein Assay Kit (Shanghai Beyotime Biotechnology Co., China).

## Author contributions

Y.-J. Jiang and S. Hui contributed equally to this work. Y.-J. Jiang: conceptualization, writing – original draft, visualization, investigation; S. Hui: investigation, methodology, validation, data curation; S. H. Tian: validation, software; Z. X. Chen: writing – review & editing; Y. F. Chai: validation; L.-P. Jiang: writing – review & editing; J.-R. Zhang: supervision, formal analysis; J.-J. Zhu: writing – review & editing, supervision, project administration, funding acquisition.

## Conflicts of interest

There are no conflicts to declare.

## Supplementary Material

NA-005-D2NA00638C-s001
