# Supplementary material for: Enhanced transmembrane electron transfer in Shewanella oneidensis MR-1 using gold nanoparticles for high-performance microbial fuel cells
Source: Nanoscale Adv. 2022 Nov 10;5(1):124–32. doi: 10.1039/d2na00638c (PMC9765428; doi:10.1039/d2na00638c)
Supplement: NA-005-D2NA00638C-s001 [file NA-005-D2NA00638C-s001.pdf]

Electronic Supplementary Material (ESI) for Nanoscale Advances.
This journal is © The Royal Society of Chemistry 2022

Supplementary Information

Enhanced transmembrane electron transfer in *Shewanella oneidensis* MR-1 by gold nanoparticles for high-performance microbial fuel cells

Supplementary Figures

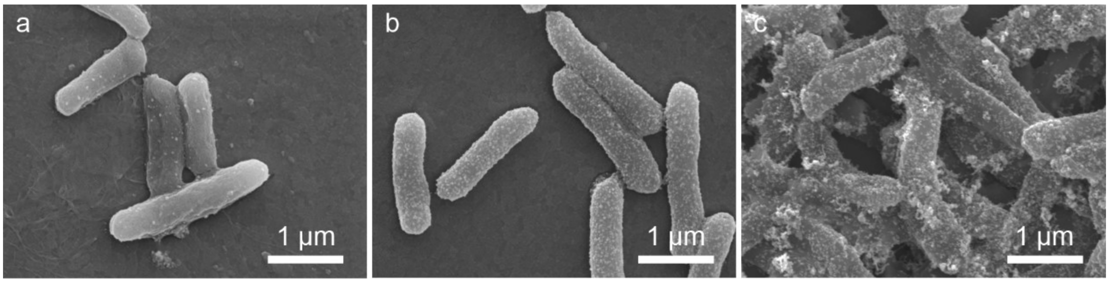

Fig. S1 SEM images of *S. oneidensis* MR-1@Au after different biomineralization time. (a) 13 h, (b) 16 h, (c) 19 h.

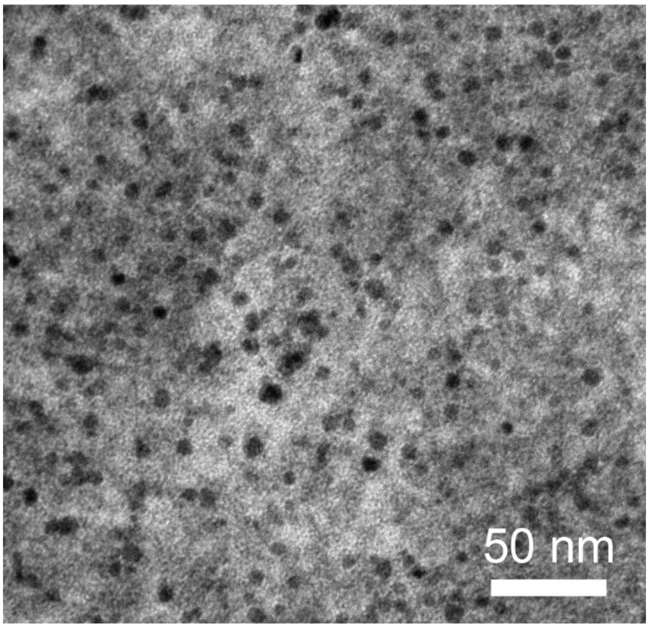

Fig. S2 TEM image of *S. oneidensis* MR-1@Au. These intracellular Au nanoparticles are about 5-10 nm in size.

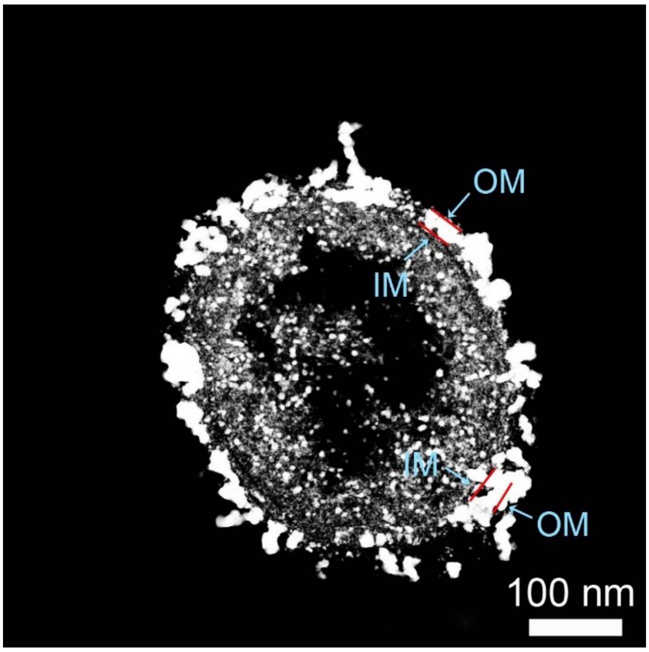

Fig. S3 high-magnification HAADF-STEM image of *S. oneidensis* MR-1@Au. OM: outer membrane of *S. oneidensis* MR-1, IM: inner membrane of *S. oneidensis* MR-1.

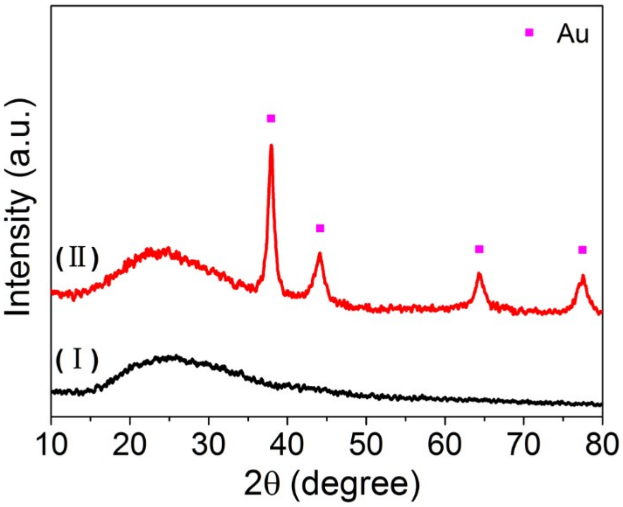

Fig. S4 XRD patterns of native *S. oneidensis* MR-1 (curve I) and *S. oneidensis* MR-1@Au (curve II).

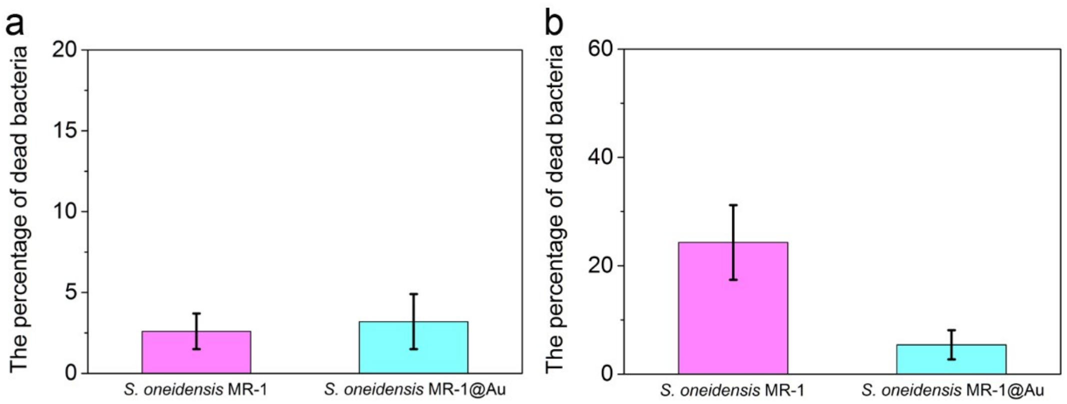

Fig. S5 (a) The percentage of dead bacteria in *S. oneidensis* MR-1 before and after Au modification. (b) The percentage of dead bacteria in *S. oneidensis* MR-1 and *S. oneidensis* MR-1@Au after 120 h aerobic incubation in medium.

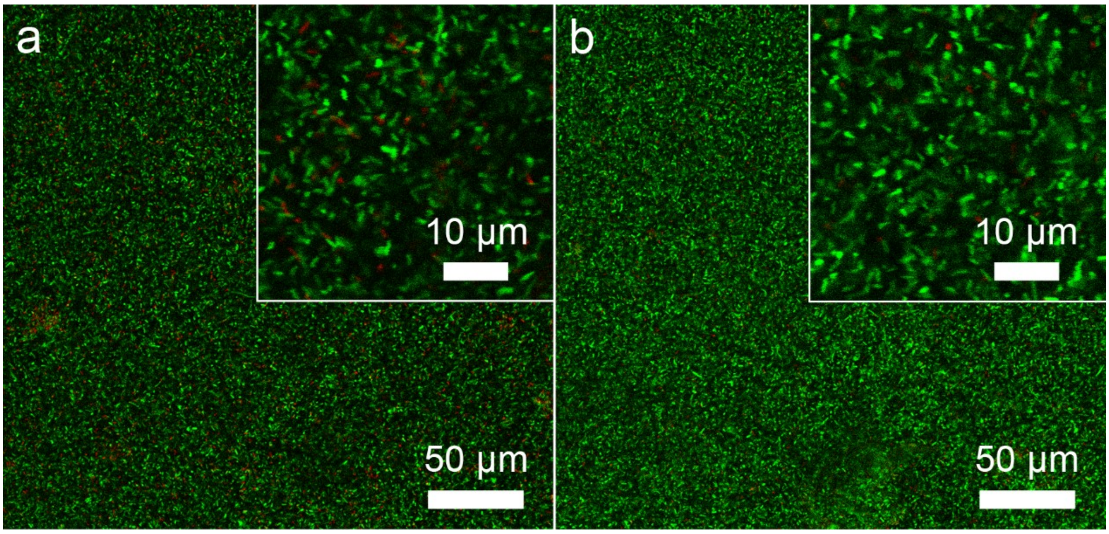

Fig. S6 CLSM images of *S. oneidensis* MR-1 (a) and *S. oneidensis* MR-1@Au (b) after cultivation for 120 h. The living *S. oneidensis* MR-1 cells were stained with a LIVE/DEAD BacLight kit, which consists of green fluorescent SYTO 9 (live cells) and red fluorescent propidium iodide (dead cells).

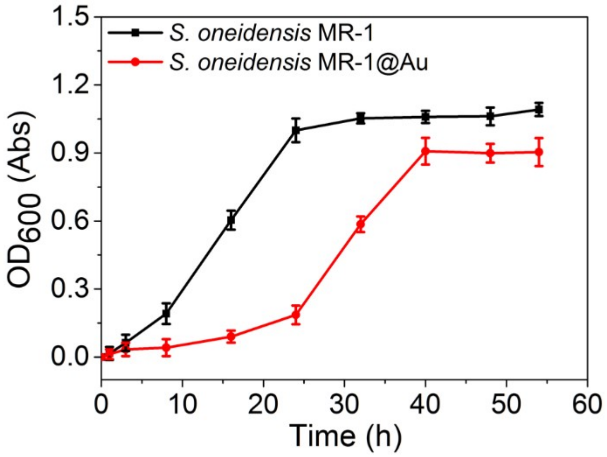

Fig. S7 Growth curves of native *S. oneidensis* MR-1 and *S. oneidensis* MR-1@Au under aerobic condition. Error bars represent standard error (s.e.) determined by three independent experiments.

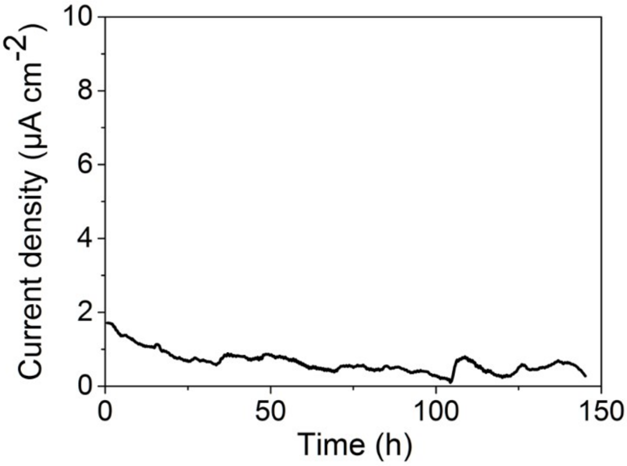

Fig. S8 Time profile of electricity generation of the electrochemical half-cell with dead *S. oneidensis* MR-1@Au.

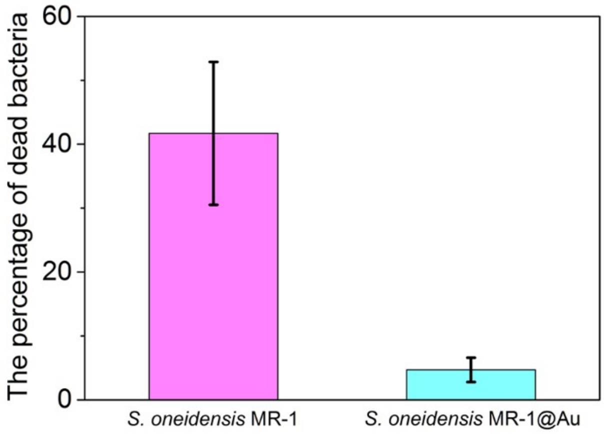

Fig. S9 The percentage of dead bacteria on the surface of *S. oneidensis* MR-1 and *S. oneidensis* MR-1@Au bioanode after anaerobic incubation in the three-electrode system for 120 h.
